# Napelline in Facial Neuralgia

**Published:** 1887-05

**Authors:** 


### Napelline in Facial Neuralgia.

M. Grognot reports the following case of facial neuralgia in which napelline produced very successful results. On January 20, 1882, the author was called to see a young girl suffering from severe pain extending over the entire head, which felt, she said, "as if it were pressed in a vice." The pain, however, was greatest on the right side in the parietal and frontal regions, and immediately above and below the eye it attained its maximum intensity. There were no tears, but, according to the patient, there had been in previous attacks. The tongue was slightly coated, but there was no caries of the teeth on the side affected. The author prescribed one granule, containing 2½ milligrammes of napelline, every two hours. On the first day the patient took ten granules; during the evening the pain disappeared. Although the pain had not returned the next morning, the patient was advised to take four granules and two the following day. Two months later the patient's neuralgia reappeared, and she took eight granules. The next day he found the neuralgia had disappeared, and since that time the health of the patient has been excellent. Two reasons caused the author to publish his observation: first, the oblivion in which this excellent medicine seems to have fallen; second, the fact that napelline may succeed where crystallized aconitine fails. In the above case the latter medicine had been administered without success, while napelline had produced complete and durable results. The author attributes the superior efficacy of amorphous aconitine over the crystallized drug to the presence of napelline in the former.—*Med. and Surg. Rep.*

